# Prognostic value of maximum standard uptake value, metabolic tumour volume, and total lesion glycolysis of 18F-FDG PET/CT in patients with malignant pleural mesothelioma: a systematic review and meta-analysis

**DOI:** 10.1186/s12935-022-02482-5

**Published:** 2022-02-03

**Authors:** Weibo Wen, Dongyuan Xu, Yongnan Piao, Xiangdan Li

**Affiliations:** 1 Department of Nuclear Medicine, Yanbian University Hospital, Yanji, Jilin Province China; 2 Center of Morphological Experiment, Medical College of Yanbian University, Yanji, Jilin Province China

**Keywords:** SUVmax, MTV, TLG, Malignant pleural mesothelioma, PET/CT, Meta-analysis

## Abstract

**Purpose:**

Present work systematically reviewed relevant literature based on 18F-FDG PET parameters and conducted a meta-analysis to examine the prognostic value of maximal standard uptake value (SUVmax), total lesional glycolysis (TLG), and metabolic tumour volume (MTV) in the prognosis of malignant pleural mesothelioma (MPM).

**Methods:**

The relevant literature published in English were searched on PubMed, Cochrane Library, and EMBASE databases. We also evaluated the significance of SUVmax, TLG, and MTV in prognosis prediction using pooled hazard ratios (HRs).

**Results:**

The current study comprised 12 primary studies with a total of 1307 MPM cases. According to our results, the pooled HR (95% confidence interval [CI]) of increased SUVmax for overall survival (OS) was 1.30 (95% CI 1.13–1.49, *P* = 0.000), whereas the increased TLG was 1.81(95% CI 1.25–2.61, *P* = 0.089). The increased MTV was not significantly related to OS (1.14 [95% CI 0.87–1.50, *P* = 0.18]).However, study design-stratified subgroup analysis suggested that differences in OS of retrospective and prospective subgroups were statistically significant, and no significant heterogeneity among different studies was observed.

**Conclusion:**

Based on the findings from the present work, PET/CT can significantly affect the prognosis prediction in MPM cases. Also, the increased SUVmax and TLG values predict an increased risk of mortality.

**Supplementary Information:**

The online version contains supplementary material available at 10.1186/s12935-022-02482-5.

## Introduction

Malignant pleural mesothelioma (MPM) is an uncommon and aggressive cancer derived from mesothelial cells.MPM is most commonly observed in menolder than 60 years, and its prognosis is poor [1–3]. MPM occurrence is high among the mesothelioma subtypes and is a refractory disorder [4, 5]. MPM is usually diagnosed at advanced stages, and palliative systemic antitumor care is preferable to aggressive surgery [6]. Immune checkpoint inhibitors (ICIs) immune checkpoint molecules are expressed physiologically on immune cells and play a key role in maintaining immune homeostasis and ensuring self-tolerance by mediating signals to attenuate excessive immune activation [7]. Immune checkpoint inhibitors based immunotherapy has been investigated in several clinical studies [8, 9] and could be an extremely effective MPM treatment [10]. The diagnostic techniques and treatments for MPM have progressed substantially [11]. Although prognostic factors such as sarcomatous histological type, sex, and performance status have been described in MPM patients [12], the imaging tool to accurately assess MPM survival and the prognostic outcome is lacking [11]. The overall survival (OS) of MPM patients is as low as 12 months [13]. Additionally, the 5-year survival for patients with MPM is extremely low. Identification of biomarkers to predict MPM prognosis for improving the clinical effectiveness of treatments is therefore crucial. Many models have been constructed for predicting MPM prognosis, including the models established by Cancer and Leukaemia Group B (CALGB) and the European Organization for the Research and Treatment of Cancer (EORTC) [14–16]. Many studies have supported that 18-fluorodeoxyglucose (18F-FDG) positron emission tomography (PET/CT) is a valuable tool for efficiently predicting and assessing cancer and the TNM stage. Mainly, FDG parameters like total lesional glycolysis (TLG), tumour volume/metabolism, metabolic tumour volume (MTV), and maximal standard uptake value (SUVmax) have been studied extensively, MTV represents the size of tumor tissue that actively ingests ^18^ F-FDG and TLG is the median SUV value in the region of interest of MTV [17–21].

Nonetheless, MPM survival prediction using the 18F-FDG PET/CT parameters is debatable.Some reports suggest that the increased SUVmax is related to the dismal survival of MPM cases [22–24], where as Doi et al. [25] did not observe such relationships. Hence, through the current meta-analysis, we aimed to evaluate the significance of SUVmax, TLG, and MTV in predicting MPM survival.

## Materials and methods

### Registration

We prospectively registered the present systematic review and meta-analysis with the PROSPERO International Prospective Register of Systematic Reviews (PROSPERO identifier CRD42020168599) [26]. The current work comprises data from previously published studies, and hence patient consent or ethical approval was waived off.

### Inclusioncriteria and literature source retrieval strategy

Cochrane Library, PubMed, and EMBASE databases were searched from 2006 to May 2021 by adopting the following keywords: ‘pleural mesothelioma’ OR ‘mesothelioma’ OR ‘malignant mesothelioma’ OR ‘malignant pleural mesothelioma’ OR ‘MPM’ AND ‘ positron emission tomography-computed tomography’ OR ‘positron emission tomography’OR ‘positron emission tomography-computed tomography’ OR ‘PET-CT’ OR ‘PET’OR ‘PET CT’ OR ‘PET/CT’ OR ‘fluorodeoxyglucose’ OR ‘FDG’ AND ‘prognosis’ OR ‘prognostic’ OR ‘outcome’ OR ‘survival’ OR ‘predictive’.

Studies with the following criteria were included: (1) MPM cases confirmed by histological diagnosis; (2) 18F-FDG PET/CT selected as pre-treatment imaging method; (3) reports with one or more than one survival data type; and (4) studies published in English. Studies with the following criteria were excluded: (1) articles focusing on diagnosis and stage alone with a disease relapse or development; (2) articles involving relapsed disorder pre-treatment; and (3) case reports, reviews, editorial materials, or conference abstracts.

Relevant studies were retrieved and selected by 2 reviewers following the specific criteria, and any disagreement between them was settled by mutual negotiation.

### Statistical analysis

To carry out the current study, we used the same method used in our previous work [27]. OS represented the duration between the start of treatment and death due to any cause. We adopted HRs and the corresponding 95% CIs for data combination and measurement of ^18^F-FDG PET parameter effect on the patient outcomes based on the HR effect size o determine the relationship among SUVmax, TLG, and MTV values with MPM survival. HR > 1 implied poorer survival whereas HR < 1 implied a survival benefit in patients with high SUVmax, MTV, or TLG. *I*^*2*^ statistic and chi-square Q tests were used to measure statistical heterogeneity, where *P* < 0.05 indicated heterogeneity. We adopted a random-effects model, whereas *I*^2^ > 50% suggested no heterogeneity and utilized a fixed-effects model. Statistical analysis was carried out using the STATA (version 12.0; STATA Corp., College Station, TX) and RevMan version 5.3 (The Nordic Cochrane Centre, The Cochrane Collaboration). We assessed for any bias through Egger’s and Begg’s tests using the STATA version 12.0. *P* < 0.05 indicated statistical significance. If both Egger’s and Begg’s tests indicat possible publication bias, trim and fill analysis would be conducted to ensure the reliability of combined HR.

## Results

### Search results

**Fig. 1:**
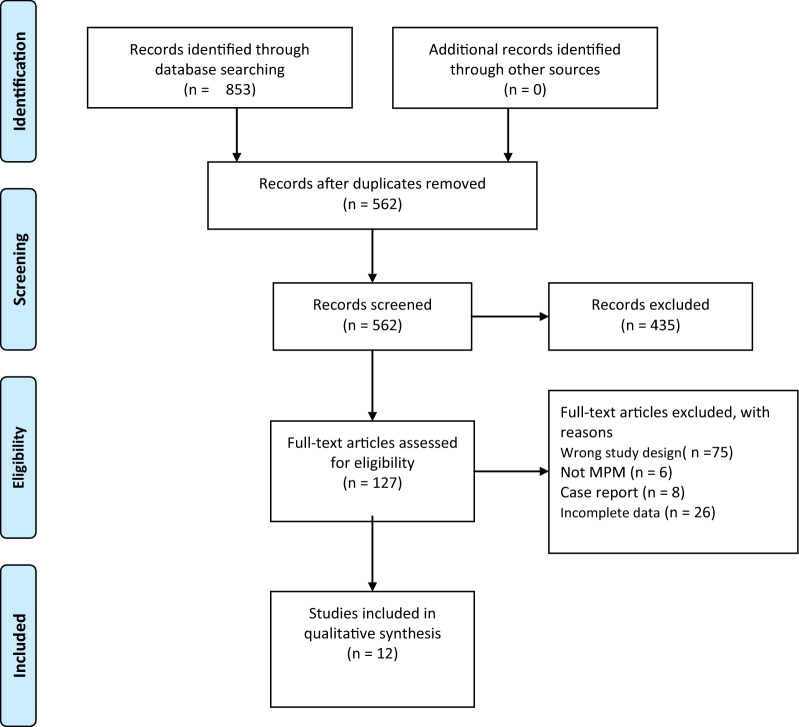
Flow diagram depicting the study selection

Figure 1 illustrates the procedure used for literature retrieval from 3 databases. Initially, 853 studies were enrolled, including 398 from PubMed, 455 from EMBASE, and 0 from Cochrane Library. Finally, 12 articles involving 1307 cases, which conformed to our pre-set selection criteria, were included in the current analysis. The enrolled articles were published between 2006 and 2021 [22, 23, 25, 28–36] (Fig. 1). All the 12 articles mentioned the significance of SUVmax, TLG or MTV in predicting MPM prognosis.

### Data extraction

Two authors collected relevant data from the enrolled articles independently (Table 1), including (1) baseline study characteristics, such as publication year, first author, time of study implementation, study design, and follow-up period; (2) patient and tumour characteristics, like a case number, median age, TNM stage, histology, endpoint, and treatment measures. In addition, we collected parameters like pre-injection fasting period, pre-injection blood glucose level, the truncated interval for the infection dose of FDG, and scanning data of 18F-FDG PET, along with the truncated value of PET parameters including SUV Max, TLG, MTV, and tumour profiles.

**Table 1:** Characteristics of included studies

Study	Year	Country	Study period	Follow-up duration (months)	Median age (range), years	No.of patients	TNM staging	End points	Study design	Histology	Treatment
Raja M. Flores et al.[36]	2006	USA	1998–2005	24	67 (35–85)	137	0–4	OS	P	Epithelioid Mixed Sarcomatoid	Surgery
Adem Koyuncu et al. [28]	2015	Turkey	2008–2012	50	53.6 ± 10.6	60	1–4	OS	R	Epithelial Biphasic Undifferentiated	Multimodality therapy Chemotherapy Best supportive care
C E Hooper et al.[31]	2015	UK	2008–2011	12	65–80	73	1–4	OS	P	Epithelioid Biphasic Sarcomatoid	Chemotherapy
Andrea Billé et al.[29]	2016	USA	2000–2013	1.2(0–68.2)	71 (46–90)	191	1–4	OS	R	Epithelioid Biphasic Sarcomatoid Non-specified	Chemotherapy Radiotherapy Palliative
Ozlem Ozmen et al.[28]	2016	Turkey	2009–2013	2.7–4.7	56.2 (28–80)	51	1–4	OS	R	Epithelial Biphasic Sarcomatoid Unidentified	Best supportive care Chemotherapy Multimodality therapy Pleuropneumonectomy Pleurectomy/decortication
David O. Hall et al.[32]	2017	UK	2008–2011	30	69–80	73	1–4	OS	P	Epithelioid Sarcomatoid Biphasic	chemotherapy
Kazuhiro Kitajima et al. [29]	2017	Japan	2007–2014	15(1–96)	66(31–84)	201	1–4	OS	R	Epithelial Sarcomatoid Biphasic Desmoplastic Others	Non Surgery Group Chemotherapy Chemotherapy and RT
Berna Akıncı Özyürek et al. [30]	2018	Turkey	2006–2014	60	56.1 ± 11.4	73	1–4	OS	R	Epithelial Biphasic	Chemotherapy Chemotherapy-Pallative radiotherapy chemotherapy
Hiroshi Doi et al. [25]	2019	Japan	2006–2015	24	68(31–84)	188	1–4	OS	R	Epithelioid Non-epithelioid	Chemotherapy radiotherapy
Filippo Lococo et al. [23]	2020	Italy	2009–2018	84	69 ± 9	141	1–4	OS	R	Epithelial Biphasic Sarcomatoid	Surgery Chemotherapy
Jun Hyeok Lim et al.[33]	2020	Korea	2009 -2018	8.7(3.8–21.9)	64 (53–71)	54	1–4	OS	R	Epithelioid Sarcomatoid Biphasic NOS	Surgery Chemotherapy
Bülent Mustafa Yenigün et al. [22]	2021	Turkey	2008–2018	13 (4–55)	60 (39–84)	65	1–4	OS	R	Epithelioid Sarcomatoid Biphasic Malignant pleural mesothelioma	Chemotherapy surgery

*NA* not available, *R* retrospective, *P* prospective, *OS* overall survival, *RT* radiotherapy, *ET* endocrine therapy

### Study characteristics

Of the 12 studies, Seven were carried out in Asia, namely Turkey (4), Japan (2), and South Korea (1), whereas other studies belonged from the USA (2), UK (2), and Italy (1). Eight retrospective and 3prospective studies were included in the current meta-analysis. Articles on 11 SUVmax treating OS as the prognostic outcome, the threshold of SUV was 2.5–10.6. MTV and TLG were measured in 6 and 7 studies, respectively, using OS as a prognosis. In addition, data including subject age at which the pathological stage of tumour was followed up were also collected. Table 1 displays study characteristics, treatment, and histology. Almost all the patients exhibited epithelioid, sarcomatoid, biphasic, mixed, and unidentified pathologies. All the studies contained at least one treatment like surgery/chemotherapy (CMT) or radiotherapy (RT).

### Literature quality evaluation was included

**Fig. 2:**
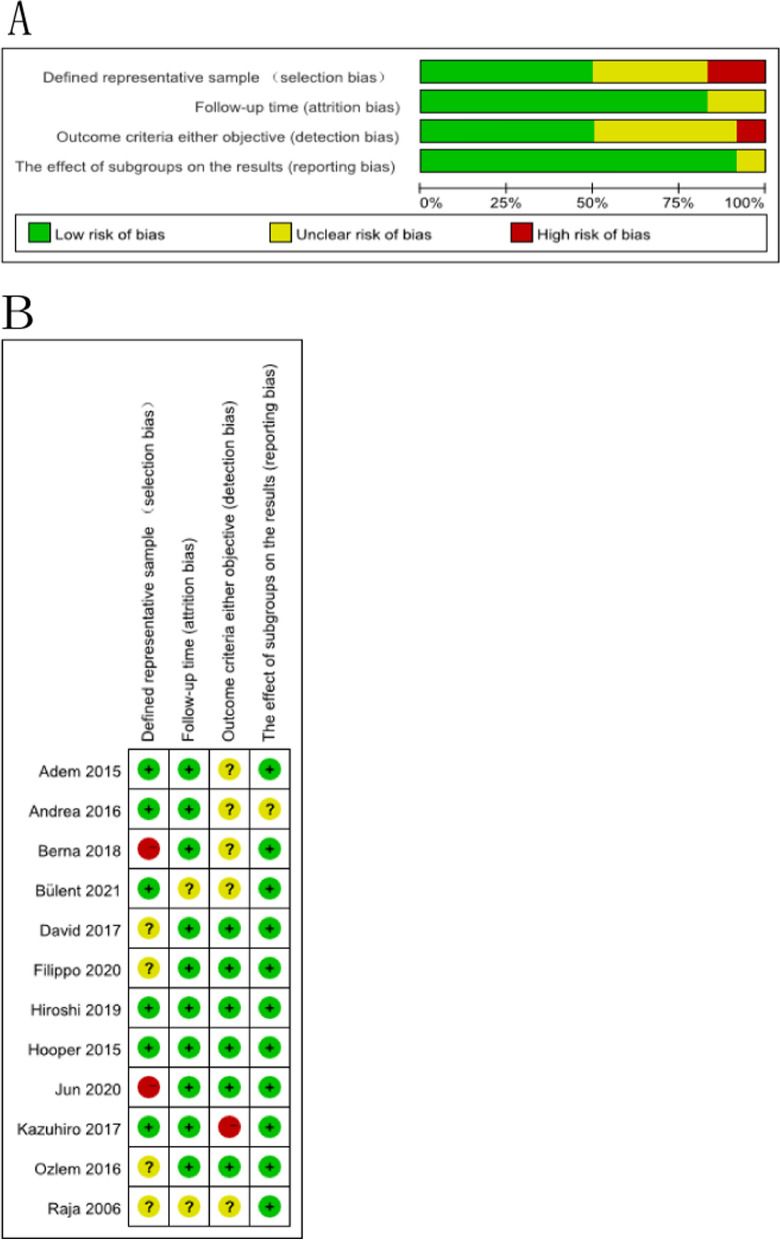
**A** Graph showing the bias risk:judgments on all risk of bias items by the reviewersdisplayed in percentageamong the enrolled articles. **B** Summary of the risks of bias:Judgment on all risk of bias items by the reviewersamong the enrolled articles

Guidelines from Critical Appraisal of Prognostic Studies (https://www.cebm.net/wp-content/uploads/2018/11/Prognosis.pdf) were applied to assess the study quality (Fig. 2). Studies enlisted in the present analysis were of high quality; however, 6 studies had an unclear or high risk of bias because of the low sample size. Meanwhile, these 6 articles with an unclear or high risk of bias in outcome criteria or objective measurements because of partial data loss of several details. Two articles illustrated an increased bias risk during follow-up time measurements because the follow-up period was short or the follow-up data was missing. Most articles were well described, and the side effects were monitored using objective standards.

### Primary outcome: OS

**Fig. 3:**
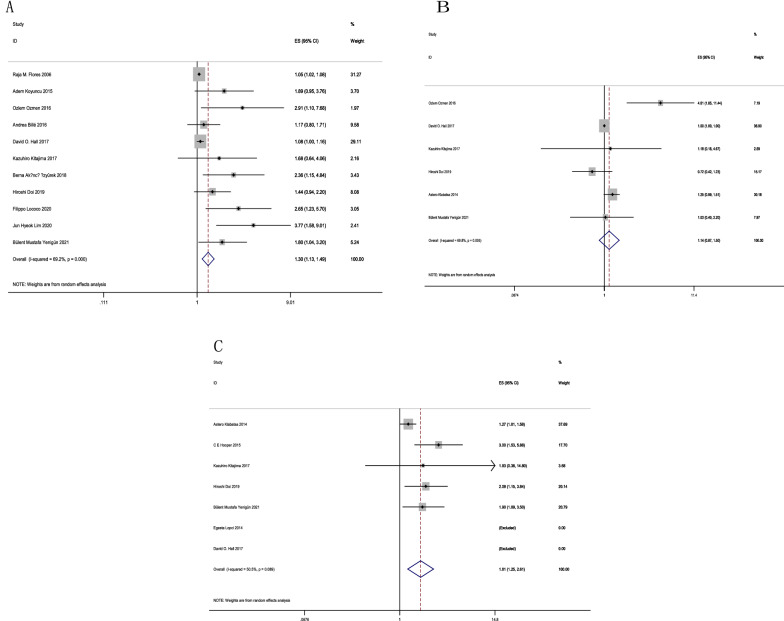
Forest plots of HR for OS with SUVmax (**A**), MTV (**B**, EFS), and TLG (**C**). Chi-square test measures heterogeneity. *P* < 0.05 indicated obvious heterogeneity. Horizontal lines = 95% CIs. Squares = estimates of single study points. Random: random-effects model.Rhombus = summarized estimate as well as the corresponding 95% CI

**Fig. 4:**
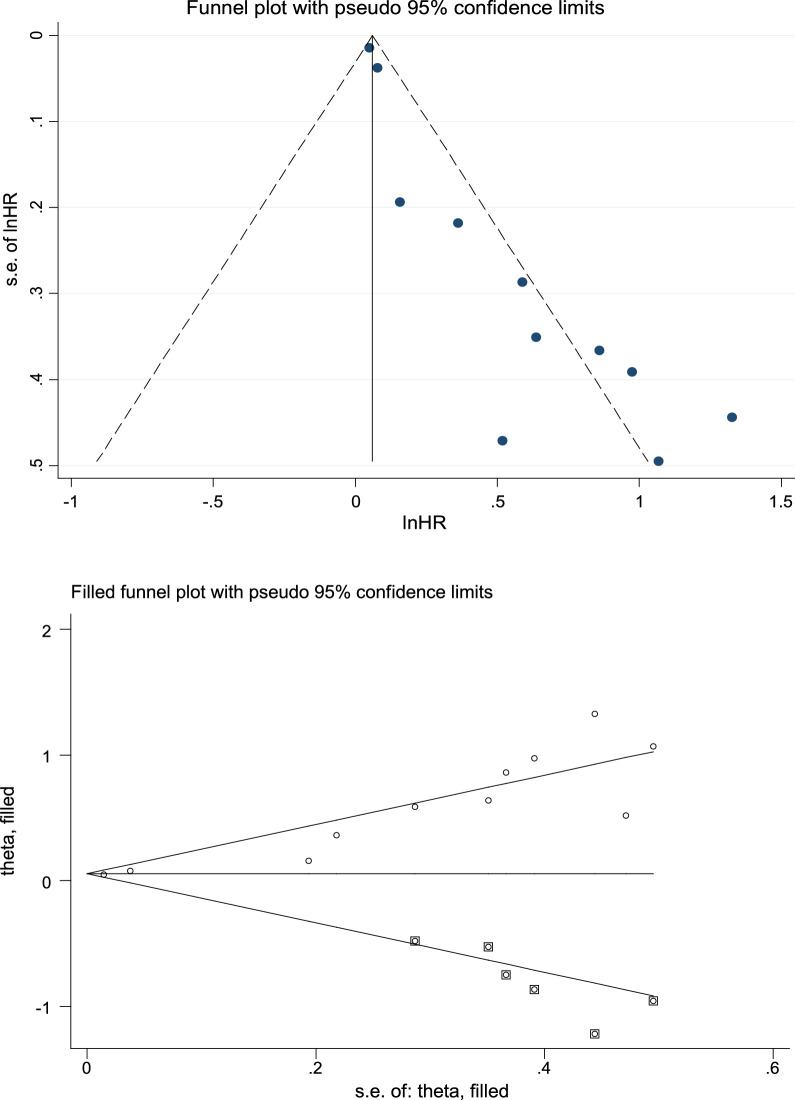
Funnel plots with (bottom column) and without (upper column) trimming and filling. The pseudo-95% CI WAs were calculated to draw the funnel plots and the related 95% CI for specific standard error. HR stands for hazard ratio

We obtained OS from 11 articles, which included SUVmax. Based on the integrated analysis, the increased SUVmax predicted poor OS, as suggested by the random- (HR = 1.30; 95% CI 1.13–1.49, *P* = 0.000) and the fixed- (HR = 1.06; 95% CI 1.03–1.09, *P* = 0.000; *I*^2^ = 69.2%) effects models (Fig. 3A). Funnel plots revealed publication bias (Fig. 4), assessed through Egger’s and Begg’s tests. *P* = 0.000 was obtained from Egger’s test, whereas *P* = 0.008 was obtained from Begg’s test (Additional file 2: Figure S2A), indicating possible publication bias. As a result, we conducted trimming and filling to ensure pooled HR reliability and acquired symmetrical funnel plots later (Fig. 4). Symmetrical funnel plots were obtained after trim and fill analysis, no significant change in results was observed (HR = 1.056; 95% CI 1.029–1.084) (Fig. 0.4), before and after hypothesis literature was added, indicating a significant correlation between SUVmax and OS. We carried out a sensitivity analysis for estimating the influence of pooled HRs. Excluding a single study exhibited no difference to the pooled results, demonstrating the stability of our results. We also conducted subgroup analyses based on study design, threshold, and cut-off method (Table 2). Following the study design, we obtained HR for 2 prospective articles as 1.05 (95% CI 1.03–1.08, *I*^*2*^ = 0.0%, *P* = 0.487) and that for 9 retrospective studies was 1.69 (95% CI 1.39–2.07, *I*^*2*^ = 26.9%, *P* = 0.205).Among the reports with OS, 2 used cut-off method by receiver operating characteristic (ROC), and the HR was 2.58 (95%CI 1.37–4.86, *I*^*2*^ = 35.8%, *P* = 0.212). Reports adopting cut-off method based on additional approaches, the HR was 1.22 (95% CI 1.07–1.38, *I*^*2*^ = 65.8%, *P* = 0.003). The threshold groups were divided into 2 subgroups based on median SUVmax: high (≥ 8.1) and low (< 8.1).Subgroup analysis revealed that the high threshold of HRs for SUVmax was 1.14 (95% CI 1.01–1.30, *I*^*2*^ = 69.9%, *P* = 0.005); whereas, the low threshold of HRs for SUVmax was 1.80 (95% CI 1.35–2.39, *I*^*2*^ = 0.0%, *P* = 0.619).

**Table 2:** Subgroup of OS of SUV max

End point	Volumetric parameters	Factor	No. of studies	Heterogeneity test (I^2^, P)	Effect model	HR	95%CI of HR	Conclusion
OS	SUV max	Study design						
		P	2	0.0, 0.487	Fixed	1.05	1.03,1.08	Significant
		R	9	26.9, 0.205	Fixed	1.69	1.39,2.07	Significant
		Cut off method						
		ROC	2	35.8,0.212	Fixed	2.58	1.37,4.86	Significant
		Others	9	65.8,0.003	Random	1.22	1.07,1.38	Significant
		Threshold						
		≥ 8.1	6	69.9,0.005	Random	1.14	1.01,1.30	Significant
		< 8.1	5	0.0,0.619	Fixed	1.80	1.35,2.39	Significant

*HR* hazard ratio, *CI* confidence interval, *OS* overall survival, *SUV Max* maximum standard uptake value, *R* retrospective, *P* prospective, *ROC* receiver operating characteristic

OS was analysed in 6 studies with MTV. The random-effects model was used with the combined HR being 1.14 (95% CI 0.87–1.50, *I*^*2*^ = 69%, *P* = 0.18) (Fig. 3B). These findings did not exhibit statistically significant correlations.

The present study analysed OS according to 7 articles that included TLG. Based on the integrated analysis, and increased TLG predicted a poor OS, as revealed by the random- (HR = 1.81; 95% CI 1.25–2.61) and fixed- (HR = 1.49; 95% CI 1.24–1.80, *I*^*2*^ = 50.5%, *P* = 0.089) effects models (Fig. 3C). According to Funnel plots, possible publication bias was observed (Additional file 1: Figure S1), assessed through Egger’s and Begg’s tests.

## Discussion

To our knowledge, the present meta-analysis is the first toelaborate on the significance of SUVmax, TLG, and MTV in predicting MPM prognosis.MPM is a refractory disorder with an increasing incidence worldwide [5, 37–39]. Some recent meta-analyses have verified that FDG uptake can be applied in predicting the prognosis of cancers like soft tissue sarcoma, hepatocellular carcinoma, and head-and-neck cancer (HNC) [40–45]. Prediction of OS using these parameters will certainly benefit MPM cases [46–48]. The current meta-analysis has been performed on data pooled from 12 research articles. As a result, Despite the adoption of different methods for different types of MPM patients, the increased SUVmax and TLG values predicted an increased OS risk [95% CI 1.13–1.449, *P* = 0.000)] and low HRs (1.30) [1.81 (95% CI 1.25–2.61, *P*=0.089)]. The current study suggests that MTV did not significantly predict the OS (HR=1.14 [95% CI 0.87–2.1.50, *P*=0.18], (Fig 3B) due to smaller sample size(6 reports examined OS with MTV). More studies are required for investigating the influence of MTV in predicting OS in MPM patients.

We detected heterogeneity in SUVmax for the prediction of OS (*I*^*2*^ = 69.2%; *P* = 0.000). Based on the ^18^F-FDG PET imaging protocols and guidelines, the PET/CT parameters (duration of fasting, preinjection blood glucose test, post-injection interval, and dose of ^18^ F-FDG) involved in the current work were acceptable as the values were within normal range [3, 38, 39] (Table 3). To investigate heterogeneity's potential source, subgroup analyses stratified by study design, threshold, and cut-off methods were performed on OS. First, prospective studies provide high-level evidence by evaluating the clinical endpoints and using the most efficient and reliable method. In subgroup analyses performed according to study design, the OS (1.05) (95% CI = 1.03–1.08, *I *^*2*^ = 0.0%, *P* = 0.487) of the Pro group showed statistical significance, and no statistical heterogeneity existed between studies. In contrast, retrospective studies provide relatively low-level clinical evidence due to a potential selection bias. In subgroup analyses performed according to study design, the OS (1.69) (95% CI = 1.39–2.07, *I*^*2*^ = 26.9%, *P* = 0.205) of the retro group also showed statistical significance, and no statistical heterogeneity existed between studies. Thus, data from both prospective and retrospective subgroups support our results. Second, data were further classified using cut-off method as 2 subgroups, where ROC group exhibited homogeneity (*I*^*2*^ = 35.8%, *P* = 0.212). Third, different optimal thresholds were observed in the enrolled reports; as a result, studies were classified as 2 groups, and the median was 8.1. Later, subgroup that had the threshold less than 8.1 was considered homogeneous (*I*^*2*^ = 0.0%, *P* = 0.619). Therefore, the study design, cut-off method, and threshold were considered sources of OS heterogeneity. The subgroup with a threshold above 8.1 revealed the existence of a statistically significant heterogeneity (I^2^ = 69.9%, *P* = 0.005).The current study failed to determine the threshold for prognostic SUVmax. The articles applied different cut-off values, which possibly affected the prediction of survival and occurrence of the disease. Further research is required for determining standard thresholds for prognosis prediction based on SUVmax.

**Table 3:** Methods of 18 F-FDG PET imaging of the included studies

Study	Duration of fasting	Preinjection blood glucose -test	Post-Injection interval	Dose of ^18^F-FDG	Pet parameters	Determina tion of cut- off values	Cut-off values		
							SUV	MTV(cm ^3^)	TLG
Raja M. Flores et al. [36]	6 h	NA	45	> 10mci	SUVmax	Others	10		
Adem Koyuncu et al. [28]	NA	NA	NA	NA	SUVmax	Others	8		
C E Hooper et al. [31]	6 h	Normal range	90	400 MBq	TLG	Others			1800
Andrea Billé et al. [23]	NA	NA	NA	NA	suvmax	Others	8.1		
Ozlem Ozmen et al. [35]	6 h	< 150 mg/dl	60	370–555 MBq	SUVmax,MTV	Others	8.6	112	
David O. Hall et al.[32]	6 h	Normal range	90	400 MBq	SUVmax	Others	10.6		
Kazuhiro Kitajima et al.[34]	5 h	NA	60	4.0 MBq/kg	SUVmax MTV TLG	ROC	5.6	278	525
Berna Akıncı Özyürek et al.[22]	6 h	< 180 mg/dl	60	370–555 MBq	SUVmax	Others	5		
Hiroshi Doi et al.[25]	5 h	NA	60	4.0 MBq/kg	SUVmax MTV TLG	Others	5.6	270	525
Filippo Lococo et al.[23]	NA	NA	NA	NA	SUVmax	Others	2.5		
Jun Hyeok Lim et al.[33]	6 h	< 150 mg/dl	60	5 MBq/kg	SUVmax	ROC	10.1		
Bülent Mustafa Yenigün et al.[22]	6 h	< 150 mg/dl	60	296–370 MBq	SUVmax MTV TLG	Others	9.8		

*ROC* receiver operating characteristic, *SUVmax* maximum standard uptake value, *MTV* metabolic tumour volume, *TLG* total lesion glycolysis, *NA* not available

Heterogeneity in TLG for OS prediction was observed (*I*^2^ = 50.5%, *P* = 0.089). Seven articles verified that TLG was related to OS. In addition, TLG was significantly correlated with the OS, as revealed by the random-effects model. Because of the few studies enrolled, subgroup analysis was not conducted; however, the Begg’s (*P* = 0.902) and Egger’s (*P* = 0.382) tests suggested the absence of publication bias. The stability of our results was supported by sensitivity analysis.

MTV and TLG are both affected by SUV [49]. However, SUV is influenced by several patient-dependent and technical parameters, such as blood glucose levels, fasting duration and uptake duration which must be strictly controlled [3, 38, 47]. SUV and other confounders possibly influence the relation of TLG with survival, and the increased TLG were related to patient survival. However, Owing to the lack of statistical data on TLG in relation to survival, systematic analysis was not possible, this study failed to establish the best threshold for TLG. Future high-quality study design and methods could find the best threshold for TLG. Similarly, SUVs and other confounders may affect the relationship between MTV and survival. The current study suggests that MTV did not significantly predict the OS (HR = 1.14 [95% CI 0.87–2.1.50], More studies are required for investigating the influence of MTV in predicting OS in MPM patients.

The current meta-analysis has some limitations. First, our enrolled articles were assessed by the Cochrane risk bias tool, and most of them were of high quality. In addition, some of the reports did not provide adequate details about 18F-FDG PET scanning data and patients. Moreover, further investigations involving PET parameters and MPM survival data are required for more conclusive analyses. Second, the sample sizes of the enrolled reports were small (n = 1307). Third, because of MPM heterogeneity, the present meta-analysis included cases at diverse stages, histological grades or those receiving various treatments, which might have a specific influence on survival and the occurrence of events over time. Fourth, the current work did not include studies published in languages other than English, which might affect possible language bias. Fifth, we used articles published only in electronic databases, which might result in possible publication bias. Nonetheless, our result reliability was verified by evaluating the publication bias.

## Conclusion

Despite the adoption of different methods for different types of MPM patients, the present work discovered the significance of PET/CT in predicting the prognosis of MPM cases. We discovered that MPM cases exhibiting increased SUVmax and TLG had an increased risk of mortality. However, the current work failed to illustrate the significance of MTV in predicting patients’ deaths. Further large-scale prospective studies are warranted to confirm the prognostic value of PET/CT parameters in MPM patients.

## Supplementary Information

**Additional file 1: Figure S1.** Funnel plots of OS and TLG.

**Additional file 2: Figure S2.** Egger’s test for OS with SUVmax and TLG (A, SUVmax; B, TLG).

## Data Availability

Not applicable.
